# ﻿Assessment and prediction of copper release amount from copper oxide facepieces

**DOI:** 10.3389/fpubh.2025.1664838

**Published:** 2025-09-25

**Authors:** Zengqing Bai, Chenchen Sun, Jinyan Liu, Zenghui Liu

**Affiliations:** School of Engineering and Technology, China University of Geosciences, Beijing, China

**Keywords:** disposable facepiece, machine learning, ﻿release amount, exposure level, ﻿support vector machine

## Abstract

**Background:**

Disposable facepieces, as important personal protective equipment, provide respiratory protection for workers. However, Cu containing facepieces may cause Cu release, posing a potential danger to human health.

**Methods:**

In this study, aging experiments were conducted on 36 groups of facepieces, simulating the use of facepieces under high temperature, radiation environment and work rate to assess the exposure levels of workers to Cu amount. Meanwhile, a machine learning model was developed based on the Cu release amount to predict the exposure level.

**Results:**

The research found that after simulating the aging of facepieces, the Cu release ranged from 7.25µg to 23.65µg, and the release trend showed an increasing trend under the simulated harsh conditions. The exposure levels in different scenarios were evaluated based on the release amount. Among them, 27 groups were evaluated as level III and 9 groups were evaluated as level II. Furthermore, the prediction results of Support Vector Machine (SVM), Backpropagation Neural Network (BPNN), and Random Forest (RF), test and training sets were evaluated using coefficient of determination (R^2^), root mean square error (RMSE) and mean absolute error (MAE). Among them, the SVM algorithm performed the best, further improving its predictive ability by using data augmentation methods and Particle Swarm Optimization (R^2^ of 0.9045, RMSE of 0.0762, and MAE of 0.0525). The relative errors between the predicted values and the true values of all samples were mostly less than 5%.

**Conclusion:**

The research method in this study can effectively assess the Cu exposure level of workers and provide a scientific basis for occupational health monitoring.

## 1 Introduction

In recent years, with the widespread application of microbial technology, a large amount of highly polluting aerosols have been released (1, 2)﻿. Environmental disruptions, such as floods, have also been shown to significantly increase health risks, including diarrheal morbidity (3), highlighting the complex interplay between environmental and occupational hazards. The KN95 disposable facepieces have become important personal protective equipment in medical, sewage treatment and other workplaces (4). Therefore, workers need to wear facepieces for a long time to ensure respiratory health (5). Among them, copper oxide (CuO) facepieces widely sold on the market have attracted widespread attention due to strong antibacterial ability, prevention of secondary infections, and effective avoidance of respiratory and lung infections caused by inhaling bacteria and other microorganisms (6, 7). However, in workplaces such as medical and industrial fields, workers often face high temperature environments, the possibility of exposure to ionizing radiation (8, 9), and different work rates (10). The interaction of these factors may affect the structural stability of facepieces, leading to material aging (11, 12), which leads to a decrease in the bonding strength between the Cu and the textiles in the facepiece. CuO attached to the fiber is easy to separate and enter the surrounding and internal environment of the disposable facepiece (13), which may increase the exposure level of the wearer. ﻿The previous study has shown that the Cu released by facepieces can induce the production of reactive oxygen species, leading to cellular oxidative damage and posing a threat to the human respiratory system (5). When CuO is used as the component material of the whole disposable facepiece, attention needs to be paid to the cytotoxicity of the facepiece to human cells (14). It is necessary to carry out the prediction research on the Cu ﻿release amount of disposable facepiece in order to scientifically assess its health risk.

With the continuous deepening of the application of machine learning (ML) in the textile field (15, 16), ML’s ability to identify, classify and predict unknown situations through existing data (17) is forging a new technical pathway for the evaluation and forecasting of fabric material performance (18). However, the current research on the Cu exposure level in the facepiece is still insufficient, and the prediction of Cu exposure in the disposable facepiece faces the problems of insufficient automation and intelligence level and low efficiency (19, 20). Therefore, applying ML to predict the exposure level of Cu in facepieces faces two major challenges: obtaining reliable and comprehensive datasets and selecting appropriate ML models. Specifically, the determination of Cu shedding from the facepiece is influenced by multiple factors, including temperature, irradiance, and work rate, which increases the complexity of index determination. To enhance the validity and universality of the model, the testing process should be as comprehensive as possible, covering a wide range of data. To ensure the accuracy and repeatability of data, the data collection process must be highly standardized and precise, which places higher demands on the experimental procedures. Furthermore, among the numerous existing ML models, choosing a predictive model that conforms to the characteristics of the data in this study is itself a major challenge. ML methods such as Support Vector Machine (SVM) Backpropagation Neural Network (BPNN), and Random Forest (RF) can predict unknown data with limited data (15, 21, 22), effectively compensating for the limitations of traditional research.

Therefore, this study simulated harsh ﻿work environments and collected Cu using ultrasonic bath treatment (23), and characterized and analyzed release levels using inductively coupled plasma (ICP) technology. According to the exposure threshold value, the release amount is classified into levels to evaluate the exposure level of the workers. At the same time, this study screened three classic prediction algorithms: SVM, RF, and BPNN. The regression model with the best performance was selected through evaluation metrics, and the model is optimized by using data augmentation methods and particle swarm optimization (PSO) algorithm to obtain more accurate predicted values. These results not only quantify the health risks posed by Cu release from facepieces, but also leverage the ML models introduced here for the first time to build a higher-precision ﻿release amount predictive model, significantly advancing its intelligence.

## 2 Materials and methods

### 2.1 Facepiece samples

This study selected KN95 CuO facepieces that comply with the Chinese national standard GB 2626–2019 “Respiratory Protection-Non-powered air-purifying particle respirator” (24). The facepiece is designed without an exhalation valve and has a five-layer structure, with the outermost and innermost layers being non-woven fabrics containing CuO.

### 2.2 Facepiece aging experiment

In this study, the temperature, irradiance and work rate, which were the key factors affecting the release of Cu from CuO disposable facepiece, were selected, and the processing time dimension was increased. In order to explore the release of Cu containing disposable facepiece under long-time operation, and reduce the possible loss of Cu in the process of storage and transportation due to the use of filter collection, the method of collecting Cu in ﻿pure water was adopted, and the breathing energy borne by the disposable facepiece was converted into ultrasonic energy by ultrasonic treatment. The ﻿experimental levels were as follows: temperature (30 °C, 50 °C), irradiance (0.75μw/cm^2^, 1.30μw/cm^2^, 1.85μw/cm^2^) and superimposed with respiration. ﻿The level of each factor was determined according to the literature review and the actual situation. 30 °C represented the indoor working temperature, and 50 °C represented the temporary high temperature that might be encountered outdoors; According to the exposure level, 0.75µw/cm^2^ represented indoor scattered ultraviolet light, 1.30µw/cm^2^ represented low-intensity areas farther away from the light source, and 1.85µw/cm^2^ represented high-intensity areas closer to the light source. For the work rate factor, the maximum breathing energy borne by CuO disposable facepiece was included in the 8-hour working time, which was converted to ultrasonic treatment. According to the content of released Cu, the exposure level of each worker during the 8-h operation process was evaluated with the Cu released by the disposable facepiece as the dependent variable.

**Figure 1:**
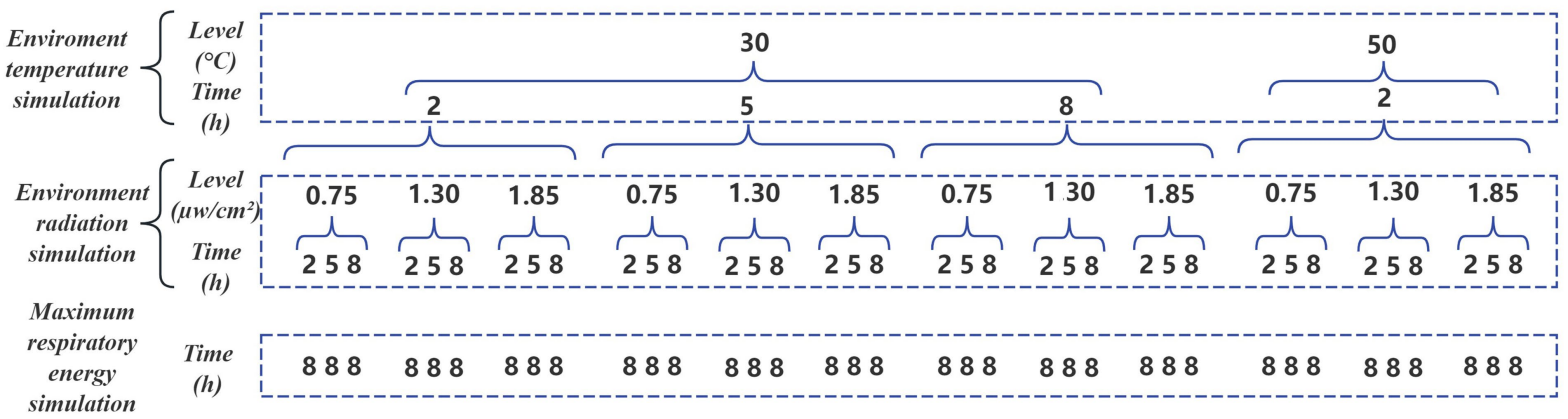
Diagram of all experimental combinations.

A total of 36 combinations were designed in the experiment to simulate the aging of facepieces in different work scenarios (Figure 1). The processing time for each experiment refers to the recommended replacement time (2 h) and maximum usage time (8 h) of the facepiece, and an intermediate node (5 h) is added to comprehensively evaluate the impact of different usage times on the performance of the facepiece.

﻿A constant temperature chamber (KBF series, BINDER GmbH, Germany) was used to simulate the workplace temperature. In order to simulate the radiation intensity in the workplace, a UV light source (TS 6 W UVA-340 nm, China) was used to simulate the UV radiation that the facepiece may come into contact with.

To simulate the effect of respiratory rate on facepieces, the ultrasonic bath ﻿treatment was used (25). The ultrasonic bath treatment ﻿used an ultrasonic cleaning machine (AK-040SD, power 480 W, capacity 10 L) to investigate the maximum respiratory energy that the facepiece can withstand. Pure water ﻿was used to collect the Cu released from the facepiece, and the processing time ﻿was calculated using Equation 1:


(1) $$t=\frac{E\times N\times T}{(P/V)\times V_{\text{mask}}\times \mathrm{\Delta t}}$$

Among them: t represents ultrasound time; E represents the energy consumption per breath, taking the maximum facepiece energy cost as 10 mJ (26); N represents the number of breaths, taking the maximum respiratory rate of 60 breaths per minute during human movement (27); T represents the total time, with a maximum wearing time of 8 h for the facepiece; P represents ultrasonic cleaner power of 480 W ; V represents the volume of the solution, the water solution used for ultrasonic treatment is about 8 L; $V_{\text{mask}}$ represents the volume of a single facepiece (9cm^3^); Δt represents time interval (1 min). The calculation result ﻿was 8.89 min. Due to the loss of ultrasonic energy caused by glass bottles, the final ultrasonic time ﻿was determined to be 9 min.

Therefore, in the process of using ultrasonic bath, the processed facepiece containing Cu was cut into approximately 2cm×2cm sheet-like shapes and placed in a wide mouthed glass bottle containing 100 mL pure water. It was then placed in an ultrasonic cleaning machine and continuously vibrated for 9 min . After the experiment, an appropriate amount of sample solution was taken into a glass sample bottle and detected using an inductively coupled plasma (ICP) technology.

To verify the feasibility of the method, a comparison was made between ultrasonic bath treatment and filter collection methods. The ﻿amount of Cu collected by ultrasonic bath ﻿treatment was 12.50 μg. The filter collection method ﻿used filter (AFT TEST MEDIA PN 813010, USA). The identical CuO facepiece was subjected to a unidirectional constant respiratory flow rate of 85 L/min for a duration of 8 h, and the Cu were collected on a filter. The ﻿amount of Cu collected was determined to be 2.13 μg upon analysis with ICP technology.

Comparing two methods, the amount of Cu collected by ultrasonic bath treatment is about 5.87 times that collected by filter collection method. Taking 95% of 5.87, the amount of Cu collected by ultrasonic bath treatment is about 5.6 times that collected by filter collection method. This estimation took into account the sinusoidal shape of the human breathing curve, suggesting that the release of Cu might increase during actual respiration. Additionally, there might have been losses when using the filter method for storage and detection processes. Overall, it was believed that within an equivalent timeframe, the ultrasonic bath treatment released about 5.6 times the amount of Cu from the facepiece compared to normal use, indicating that the ultrasonic bath treatment not only collected more material but also saved time.

The facepiece aging experiment was conducted according to the values set for each group. Initially, the facepiece was placed in ﻿the constant temperature chamber with a humidity of 85%, and then the surface of the facepiece was exposed to UV light. Subsequently, the facepiece was treated using the ultrasonic bath ﻿treatment employed in the pre-experiment. A total of 36 experiments were conducted following the instructions. The collected solutions were then labeled and stored for subsequent ICP analysis.

### 2.3 ICP analysis

The content of Cu was analyzed on ICP (ThermoICPOES7200, ThermoFisher, USA). The instrument was operated at an RF power of 1.15 kW with argon as carrier and plasma gas. The plasma flow was set to 15 L/min, the auxiliary gas flow was set to 1.5 L/min, and the nebulizer gas flow was set to 0.75 L/min; Detection was carried out in axial view mode, and linear calibration was employed. Each sample was measured in triplicate, and the average value was taken.

### 2.4 Exposure level assessment

To assess the potential exposure level of Cu that workers may face while wearing KN95 CuO facepieces, this study used the Time Weighted Average Allowable Concentration (PC-TWA) in the Occupational Exposure Limit (OEL) as the evaluation indicator﻿ (28). The Equation 2 is as follows:


(2) $$\mathrm{TWA}=\frac{C_{1}T_{1}+C_{2}T_{2}+\cdots +C_{n}T_{n}}{T_{1}+T_{2}+\cdots +T_{n}}$$

Among them: C is the contact concentration; T is the duration of contact, where $T_{1}+T_{2}+\cdots +T_{n}=8h$ . When the workers wear the facepieces, the closed space ( V ) formed between the facepieces and the human face is approximately 5 × 10^−4^ m^3^. According to the standard GB 2626–2019 (24) and the grade of facepiece filter material, the overall total leakage rate is 11%. The amount of Cu collected by the ultrasonic bath treatment is approximately 5.6 times that under normal breathing conditions. Excluding other physical processes like human inhalation, the actual $\mathrm{TW}A_{\text{worker}}$ , namely the occupational exposure limit for the worker ( $\mathrm{OE}L_{\text{worker}}$ ), is calculated using Equation 3 as follows.


(3) $$\mathrm{TW}A_{\text{worker}}=\mathrm{OE}L_{\text{worker}}=\frac{(x/8V)\times (1-11\%)\times 8÷5.6}{8}$$

Reference was made to GBZ 2.1–2019 ﻿‘Occupational exposure limits for hazardous agents in the workplace-Part 1: Chemical hazardous agents’ for occupational exposure levels and classification control tables (28), to calculate exposure limits for different levels. ﻿Let x mg be the amount of Cu detected by ICP over an 8-hour period. Considering that in the worst-case scenario, assuming that all ﻿Cu detected by ICP was ﻿Cu dust, the OEL value of cu dust was 1 mg/m^3^ (28), and the calculation results were summarized in Table 1 by substituting it into Equation 3. Based on the release of ﻿Cu from CuO facepieces in different work scenarios, the exposure level of workers in different work scenarios could be evaluated using the work exposure level table.

**Table 1:** Occupational exposure level and limit.

Exposure level	Exposure limit (μg)	Level description
0 (≤1% OEL)	≤0.25	Basically contactless
I (>1%, ≤ 10% OEL)	>0.25, ≤ 2.5	Very low contact, no relevant effect based on existing information
II (>10%, ≤ 50% OEL)	>2.5, ≤ 13	Contact but no significant health effects
III (>50%, ≤ OEL)	>13, ≤ 25	Significant contact requires action to restrict activities
IV (>OEL)	>25	Exceeding OELs

### 2.5 Construction of prediction model for cu release in disposable facepiece

#### 2.5.1 Data source and preprocessing

The Cu release prediction model constructed in this study was based on the aforementioned 36 sets of experimental data. The input features of the model were temperature, temperature exposure time, irradiation intensity, and irradiation exposure time, and the output was the corresponding release amount of Cu. The work scenario considered the worst-case scenario, so the work rate was not used as an input variable, but as a background condition. Before modeling, the data was preprocessed first. In this study, categorical variables included temperature, temperature exposure time, irradiation intensity, and irradiation exposure time; ﻿the quantitative variable was the amount of Cu released. For categorical variables, the Label Encoding method was used to map each category to a unique integer value (29) (Table 2).

**Table 2:** Factor and label encoding.

Temperature	Label encoding	Temperature exposure time	Label encoding	Radiation intensity	Label encoding	Irradiation exposure time	Label encoding
30	0	2	0	0.75	0	2	0
50	1	5	0.5	1.30	0.5	5	0.5
-	-	8	1	1.85	1	8	1

Subsequently, the Min-Max Normalization method was used to scale the values of all variables to the [0, 1] interval, in order to eliminate the dimensional influence between different variables. The Equation 4 is as follows:


(4) $$X\prime =\frac{X-X_{\min}}{X_{\max}-X_{\min}}$$

Among them: X′ represents the normalized data; X represents sample data; $X_{\max}$ and $X_{\min}$ representthe maximum and minimum values in the sample. During the model training phase, 85% of the data is used as the training set, and the remaining 15% is used as the testing set.

#### 2.5.2 ML algorithms

In recent years, ML algorithms have been increasingly applied in fields such as textile materials and protective materials, especially in prediction and classification tasks, providing several significant advantages. This study selected three mainstream ML algorithms (i.e., BPNN, RF, and SVM) to predict the release of Cu from facepieces in work scenarios. Prediction models were established for each algorithm, and evaluation metrics R^2^, RMSE, and MAE were used to compare and analyze the predictive performance of different models to determine the optimal modeling method.

##### 2.5.2.1 BPNN

BPNN is a typical multi-layer feedforward neural network that simulates the structure of the biological nervous system, typically including an input layer, one or more hidden layers, and an output layer (30–32). The basic pipeline includes two stages: forward propagation and backward propagation:

Forward propagation is a neural network that processes input information layer by layer. The output of neurons from i -th layer to j -th layer could be expressed as Equation 5 (33, 34):


(5) $$y_{j}^{m}(n)=f(\sum_{k=1}^{p}w_{\mathrm{ji}}^{k}(n)y_{i}^{k}(n))$$

Among them, $y_{j}^{m}(n)$ represents output value of the m -th neuron in the j -th layer. f(⋅) is the activation function and introduce nonlinear characteristics p : the total number of nodes in the i -th layer. $w_{\mathrm{ji}}^{k}(n)$ is the weight of the j -th neuron from the k -th layer to the m -th layer. $y_{i}^{k}(n)$ is the output value of the i -th neuron in the k -th layer at the n -th time step.

Backpropagation is the process of updating weights through error backpropagation algorithm to minimize the loss function. The weight update formula is Equation 6 (33, 34):


(6) $$w(n+1)=w(n)-\eta \frac{\partial E}{\partial w(n)}+\mathrm{\varepsilon \Delta w}(n)$$

Among them, w(n) represents the weight at the n -th iteration. η is the learning rate that controls the step size of weight updating. E is the energy of prediction error. $\frac{\partial E}{\partial w(n)}$ is the partial derivative of error with respect to weight, representing the sensitivity of error to weight. ε is a momentum parameter for accelerating convergence. Δw(n) is the momentum term of the weight, used to avoid local minima.

##### 2.5.2.2 RF

RF is an ensemble learning method that improves the stability and generalization ability by constructing multiple decision trees and performing ensemble voting on outputs. This method combines Bagging technique with feature random selection strategy, reducing the sensitivity of the model to outliers and noise (33, 35, 36). The prediction function Equation 7 (33) is as follows:


(7) $$H(x)={\text{argmax}}_{Y}\sum_{i=1}^{n}I(h_{i}(x)=Y)$$

Among them, H(x) is the final prediction result of the random forest model on the input x . ${\text{argmax}}_{y}$ represents the selection of the y value that maximizes the internal expression. $\sum_{i=1}^{n}I(h_{i}(x)=Y)$ is the sum of the predicted results of all trees, where $I(h_{i}(x)=Y)$ is an indicator function. When the $h_{i}(x)$ predicted result of the i -th decision tree model is equal to Y , the value of this function is 1, otherwise it is 0; $h_{i}(x)$ represents the i -th decision tree model. Y represents the final output of the decision tree.

##### 2.5.2.3 SVM

SVM was initially used for classification problems (33, 37). The core idea is to map the input space to a high-dimensional feature space through a nonlinear mapping function θ(x) , and construct the optimal hyperplane for regression fitting in this space using Equation 8 (33, 38):


(8) $$\gamma =\omega^{T}\theta (x)+b$$

Among them, γ is the predicted value; ω is a weight vector; θ(x) is a nonlinear mapping function that maps input data x to a higher dimensional feature space; b is a bias term. In order to obtain the optimal weight vector ω , it is necessary to minimize the regularization function and constrain it using Equation 9, 10, and 11 (33, 38):


(9) $$\min\{\frac{1}{2}\omega^{2}+C\sum_{i=1}^{N}(\xi_{i}+\xi_{i}^{\left(*\right)})\}$$

(10) $$\gamma_{i}-(\omega^{T}\theta (x_{i}))+b\leq \psi +\xi_{i}\;\;i=1,2,\ldots ,N$$

(11) $$\xi_{i},\xi_{i}^{\left(*\right)}\geq 0\;\;i=1,2,\ldots ,N$$

Among them, $\omega^{2}$ is the sum of squares of the weight vectors, representing the complexity of the model; C is a regularization parameter that controls the trade-off between model complexity and error; $\xi_{i}$ and $\xi_{i}^{\left(*\right)}$ are slack variables that allow some data points to violate constraints; N is the sample size. ψ is the approximation accuracy of the function placed on the training data sample; $\gamma_{i}$ is the true value of the i -th sample.

Finally, by introducing Lagrange multipliers $\delta_{i}$ and $\delta_{i}^{*}$ and utilizing kernel functions $K(x,x_{i})$ , the prediction function Equation 12 (33, 38) can be obtained:


(12) $$f(x)=\sum_{i=1}^{N}(\delta_{i}-\delta_{i}^{*})K(x,x_{i})+b$$

Among them, $K(x,x_{i})$ is the kernel function used to calculate the inner product of two vectors in high-dimensional space, thereby avoiding explicit calculation of the mapping function. The commonly used Gaussian kernel in SVM, also known as radial basis function, is chosen as the kernel function (39). The kernel function Equation 13 is as follows:


(13) $$k(x,x_{i})=\exp(-\frac{\|x-x_{i}\|2}{2\sigma^{2}})$$

Among them, $\|x-x_{i}\|2$ is the square of the Euclidean distance between x and $x_{i}$ ; σ is the bandwidth parameter of Gaussian kernel, which controls the width of the function.

#### 2.5.3 Evaluation

During the training and testing phases, The predictive performance of the ML model was evaluated using the coefficient of determination (R^2^), root mean square error (RMSE), and mean absolute error (MAE). These metrics can comprehensively measure the goodness of fit of the model (40). The Equation 14, 15, and 16 are as follows:


(14) $$R^{2}=1-\frac{\sum_{i=1}^{n}{\left(y_{i}-{\hat{y}}_{i}\right)}^{2}}{\sum_{i=1}^{n}{\left(y_{i}-\bar{y}\right)}^{2}}\;$$

(15) $$\text{RMSE}=\sqrt{\frac{1}{N}\sum_{i=1}^{N}{\left(y_{i}-{\hat{y}}_{i}\right)}^{2}}$$

(16) $$\mathrm{MAE}=\frac{1}{N}\sum_{i=1}^{N}|y_{i}-{\hat{y}}_{i}|$$

Among them, $y_{i}$ represents actual value, ${\hat{y}}_{i}$ represents predicted value, $\bar{y}$ represents the average of actual values. In this study, the model with the highest R^2^ and the lowest RMSE and MAE was selected as the optimal prediction model.

#### 2.5.4 Model optimization

After model evaluation, select the model with the best predictive performance. At the same time, in order to prevent overfitting, 36 data points were synthesized by adding Gaussian noise and 18 data points were synthesized by linear interpolation, resulting in a total of 90 data points. The dataset was still divided into 85% training set and 15% testing set. And utilize PSO algorithm for model parameter optimization (41).

By assuming that in a D-dimensional search space, the number of particles is *M*, the position of the i -th particle is $X_{i}$ , and the velocity is $V_{i}$ , the particle updates its position and velocity according to Equation 17 and 18.


(17) $$\begin{array}{c} v_{\mathrm{ij}}(t+1)=wv_{\mathrm{ij}}(t)+c_{1}r_{1}(t)(P_{\mathrm{ij}}(t)-x_{\mathrm{ij}}(t))\\ +c_{2}r_{2}(t)(P_{\mathrm{gj}}(t)-x_{\mathrm{ij}}(t)) \end{array}$$

(18) $$x_{\mathrm{ij}}(t+1)=x_{\mathrm{ij}}(t)+v_{\mathrm{ij}}(t+1)$$

Among them, w is the inertia weight; $c_{1}$ and $c_{2}$ are acceleration constants; $r_{1}$ and $r_{2}$ are random parameters. The acceleration constant $c_{1}$ and $c_{2}$ are both set to 2.05; Set the particle population size to 10; Perform triple fold cross validation on the training set. When the number of iterations reaches the set value or the optimal position found, and the set minimum adaptive value is met, the optimization process is done.

## 3 Results

### 3.1 Experimental results and exposure level assessment

#### 3.1.1 Cu release from CuO disposable facepiece under different environmental conditions

The analysis of Cu released from CuO disposable facepiece under different environmental conditions (including temperature and irradiance) showed that there were significant differences in the amount of Cu released from ﻿work scenarios. Work scenarios 1–8 typically exhibited a lower range of Cu release, typically between 5 and 10 μg, indicating that these groups were subjected to less harsh environmental conditions. In contrast, the ﻿os 9–36 showed a higher Cu release range, mainly between 10 and 20 μg, indicating that the deterioration of the work environment had led to an upward trend in the Cu release of the disposable facepiece.

**Figure 2:**
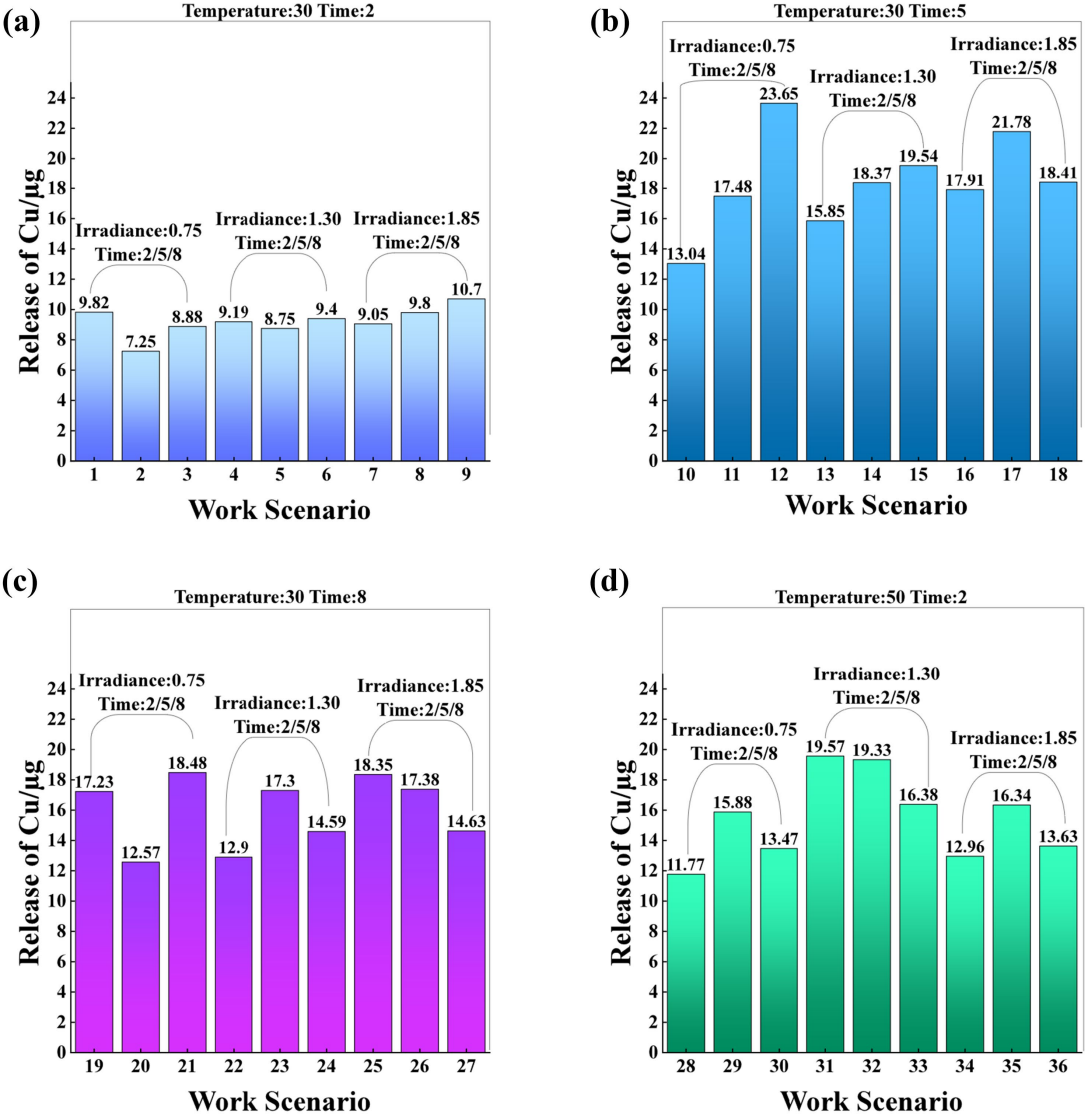
﻿The bar charts labeled **(a)** through **(d)** illustrate the Cu release under different working conditions. Each chart indicates the temperature and time. Chart **(a)** shows the copper release at 30 °C and 2 h. Chart **(b)** displays the copper release at 30 °C and 5 hours. Chart **(c)** represents the copper release at 30 °C and 8 h. Chart (d) depicts the copper release at 50 °C and 2 hours. These scenarios involve irradiance levels of 0.75 μw/cm^2^, 1.30 μw/cm^2^, and 1.85 μw/cm^2^, with irradiance times of 2 h, 5 h, and 8 h, respectively.

A particularly noteworthy observation was that the release amount of Group 12 reached 23.65 μg, the highest among all experimental groups. This may be due to a combination of higher temperatures and prolonged irradiance. The release amount of 21.78 μg in Group 17 ranked second, further indicating the influence of environmental ﻿pressure factors on the stability of CuO. The release amount of group 2 was the lowest, 7.25 μg, which indicated that the CuO disposable facepiece could be effectively stabilized at a short temperature to minimize the release of Cu. Therefore, with the deterioration of environmental conditions, the increasing trend of Cu release highlighted the key role of environmental factors in the Cu release of disposable facepiece (Figure 2)﻿ (See Table S1 in the Supplementary material).

#### 3.1.2 The influence of temperature and irradiation intensity on release ﻿trends

**Figure 3:**
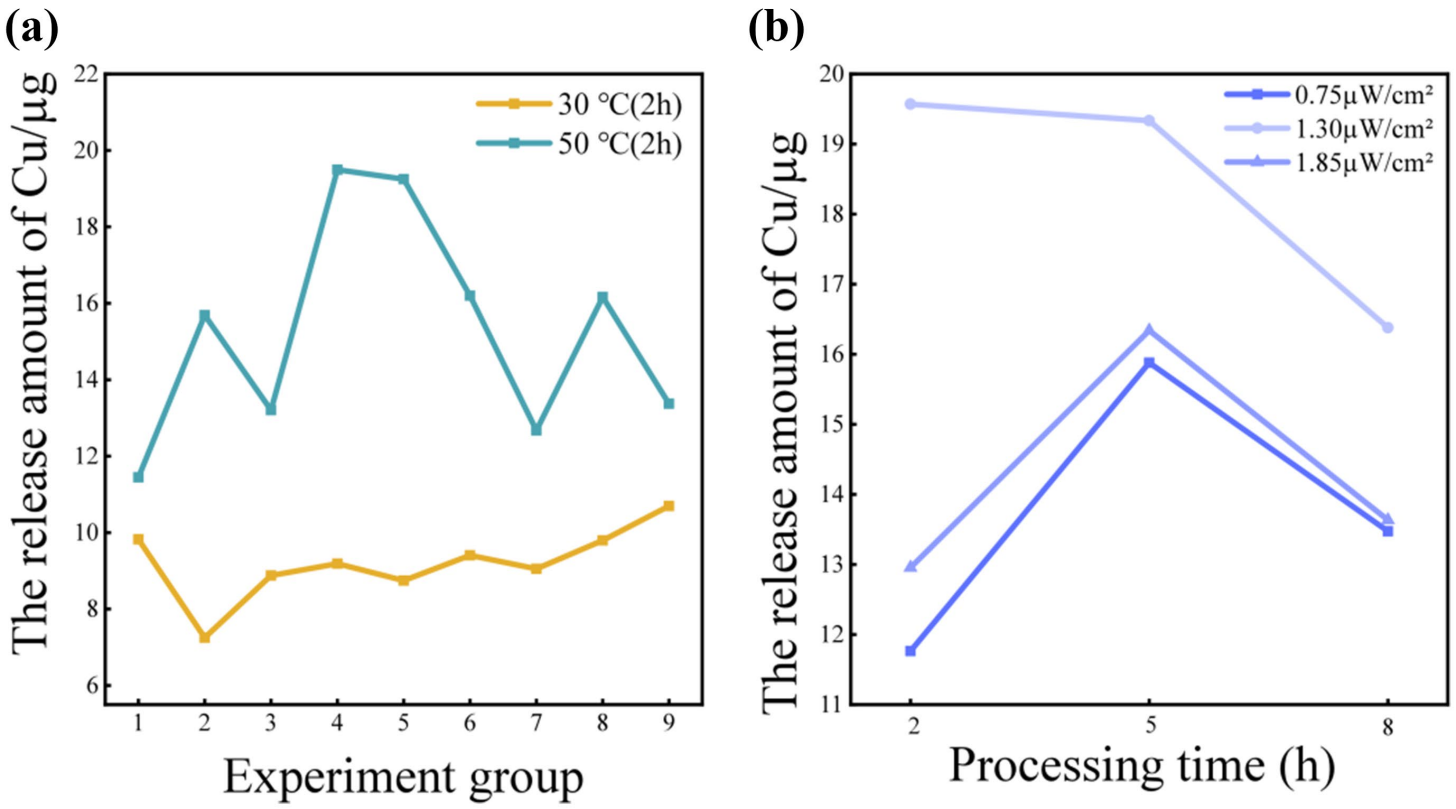
**(a)** Release of Cu at different temperatures; **(b)** Release of Cu under different irradiance.

The release of Cu from CuO facepieces at 30 °C and 50 °C was analyzed. According to the principle of controlling for a unified variable, the treatment conditions for the two groups of variables remained consistent except for temperature. The release of Cu at 50 °C was significantly higher than that at 30 °C (Figure 3﻿a), which may have been attributed to the high temperature promoting the thermal decomposition or surface reaction activity of CuO, leading to the release of more Cu.

At 30 °C, the trend of Cu changing with the radiation intensity or radiation time of each group was relatively gentle, indicating that the influence of temperature on the release of Cu should have been dominant at that time. At 50 °C, there were periodic fluctuations in the experimental data of 9 groups (Figure 3﻿a), which were due to different irradiation conditions. At this time, the effect of irradiation on the release of Cu was more significant, possibly due to the photocatalytic reaction induced by light accelerating the decomposition process of CuO (42).

Nine experiments at 50 °C were selected and divided into 3 groups according to different radiation intensities to analyze the release of Cu under different radiation intensities. At different irradiation intensities, there was a significant stratification in the release of Cu. At an irradiation intensity of 0.75μw/cm^2^, the release of Cu was at the lowest level, followed by a radiation intensity of 1.85μw/cm^2^, and the highest release of Cu was at an irradiation intensity of 1.30μw/cm^2^. The release of Cu first increased and then decreased with the increase of irradiation intensity (Figure 3﻿b).

#### 3.1.3 Occupational exposure ﻿level assessment

The occupational exposure ﻿levels of workers across different work scenarios were analyzed based on experimental data. The analysis revealed that among the 36 distinct working scenarios, a total of 27 groups of experimental workers were classified as having a level III exposure level according to the occupational exposure level and limit table (Table 1). This classification indicated a significant exposure of workers to Cu. Consequently, it was determined necessary to restrict the use of CuO facepieces in situations of long-term high-intensity exposure. The exposure levels in the remaining 9 experiments were all categorized at level II, suggesting that while there was exposure to Cu, it did not result in significant health effects

### ﻿3.2 Selection and Optimization of Prediction Model for Cu Release in Facepieces

#### ﻿3.2.1 Model performance comparison

**Figure 4:**
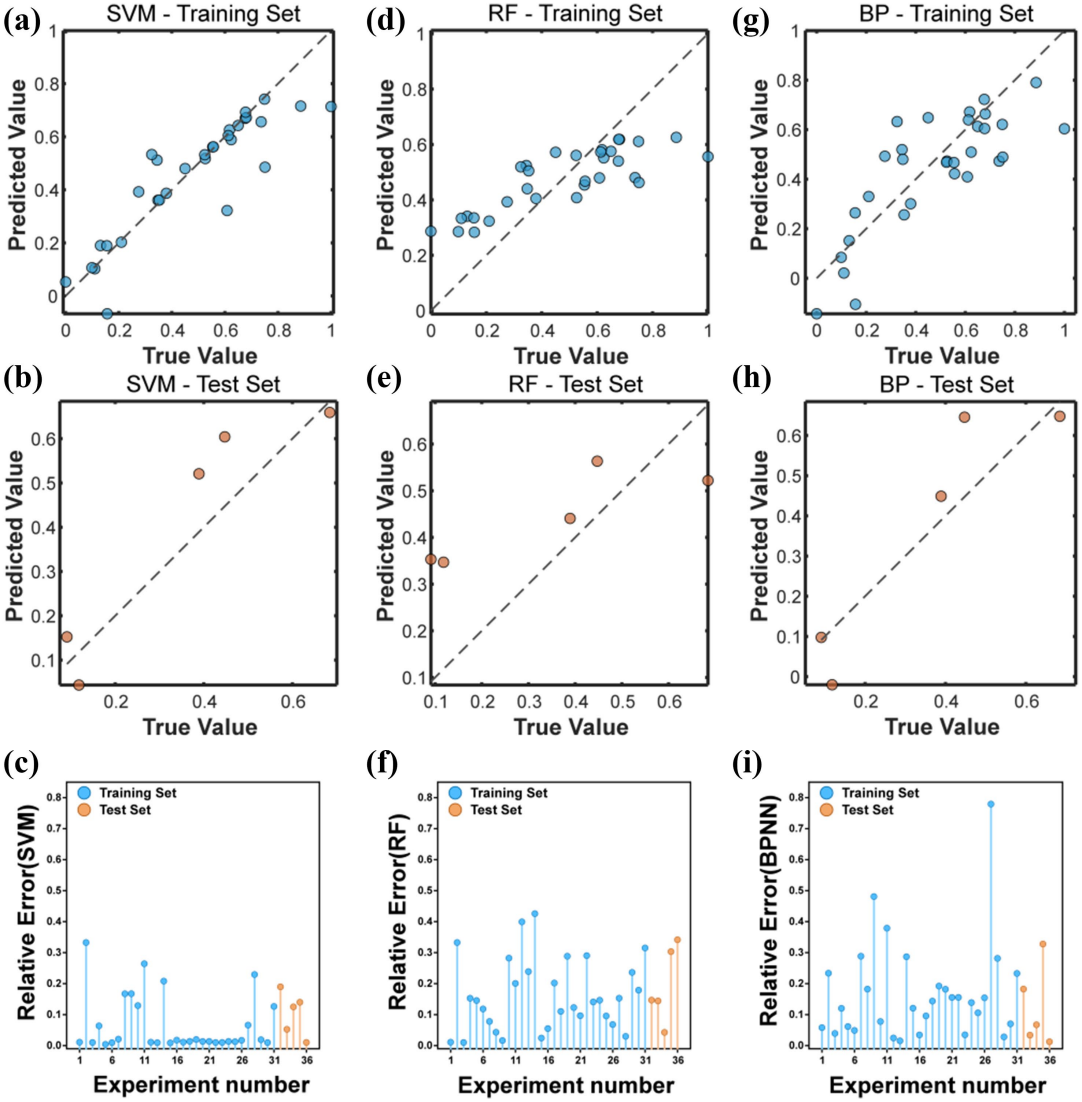
**(a,d,g)** Represent the comparison between the predicted values and the true values of SVM, RF, and BPNN on the training set. **(b,e,h)** Represent the comparison between the predicted values and the true values of SVM, RF, and BPNN on the test set. **(c,f,i)** Represent the relative errors between the predicted values and the true values of all samples for SVM, RF, and BPNN, respectively.

On the training set, the SVM model performed well, with high consistency between predicted values and true values, indicating that SVM could effectively learn from training data and make accurate predictions. The performance of BPNN model on the training set was not as good as SVM, and the consistency between predicted values and true values was poor, indicating that it had certain limitations in processing the data in this study. The distribution of points in the RF model on the training set was relatively scattered, which was still relatively poor compared to SVM. Due to the small sample size of the test, the distribution of points in the three models was relatively scattered. Overall, SVM outperformed BPNN and RF (Figure 4).

In addition, there were 26 groups of samples in the SVM model where the relative error between the predicted value and the true value was less than 10%. There were 15 groups of samples with relative errors less than 10% in BPNN, and 11 groups of samples with relative errors less than 10% in RF model, and BPNN and RF samples showed significant errors (Figure 4).

To further evaluate the performance of each model, metrics such as RMSE, MAE, and R^2^ were used. The RMSE of SVM model on the training set was 0.1159, MAE was 0.0696, and R^2^ was 0.7842. On the test set, these metrics were 0.1018, 0.0899, and 0.8422, respectively. This indicated that the SVM model had good predictive performance in the release of Cu. The RMSE of the BPNN model on the training set was 0.1593, MAE was 0.1293, and R^2^ was 0.5919. On the test set, these metrics were 0.1126, 0.0879, and 0.8071, respectively. This indicated that the BPNN model had lower predictive performance than the SVM model in predicting the release of Cu. The RMSE of the RF model on the training set was 0.1728, MAE was 0.1468, and R^2^ was 0.5202. On the test set, these metrics were 0.1808, 0.1642, and 0.5024, respectively. This indicated that the RF model had the worst predictive performance for the release of Cu in this study (Table 3).

**Table 3:** Evaluation metrics of SVM, BPNN, and RF on training and ﻿test sets.

Model	Evaluation index	Training set	Test set
SVM	RMSE	0.1159	0.1018
	MAE	0.0696	0.0899
	R^2^	0.7842	0.8422
BPNN	RMSE	0.1593	0.1126
	MAE	0.1293	0.0879
	R^2^	0.5919	0.8071
RF	RMSE	0.1728	0.1808
	MAE	0.1468	0.1642
	R^2^	0.5202	0.5024

#### ﻿3.2.2 Model optimization and improvement

**Figure 5:**
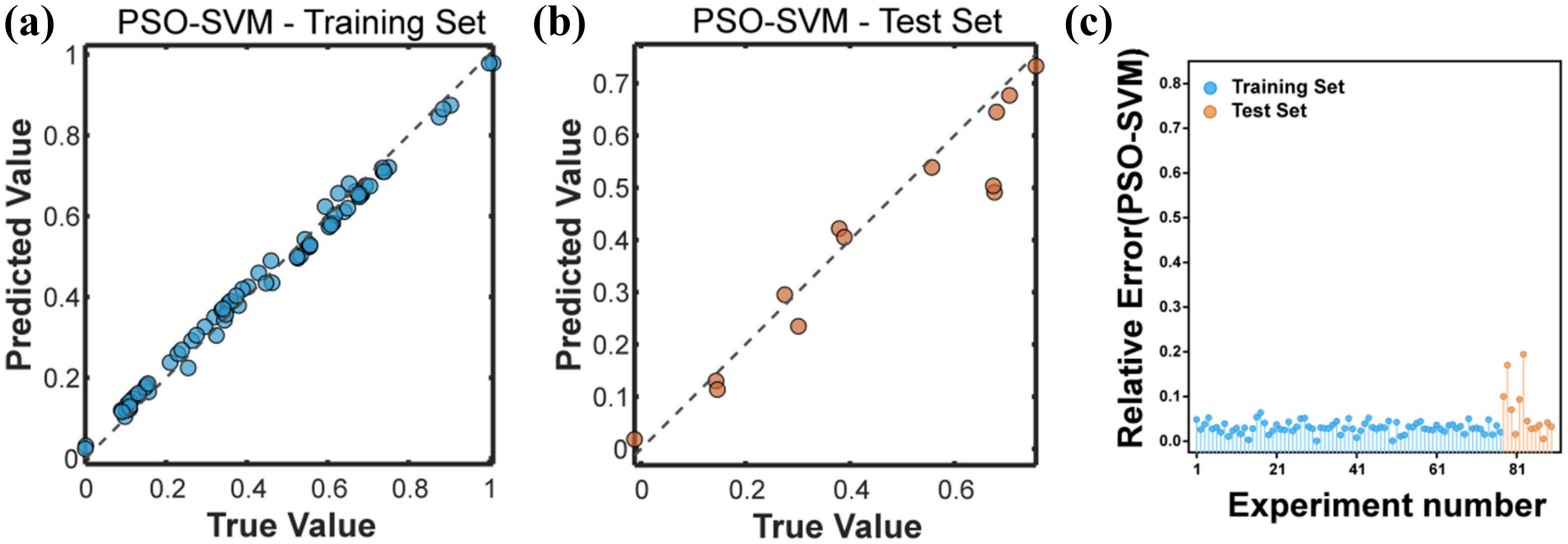
**(a)** Represents the comparison between the predicted values and the true values of PSO-SVM on the training set. **(b)** Represents the comparison between the predicted values and the true values of PSO-SVM on the test set. **(c)** Represents the relative error between the predicted values and the true values of all samples representing PSO-SVM.

Using PSO algorithm to optimize the hyperparameters of SVM model. On the training set, the RMSE of the PSO-SVM model was 0.0232, the MAE was 0.0220, and the R^2^ was as high as 0.9906. Compared with the evaluation index values of the SVM model, this indicated that PSO significantly improved the fitting degree of the SVM model for the release of Cu. On the test set, the RMSE of the PSO-SVM model was 0.0762, MAE was 0.0525, and R^2^ was 0.9045. Although the performance metrics were slightly lower than those of the SVM model on the test set, the R^2^ value was still high, and the RMSE and MAE values were low (Table 4), indicating that the model had strong generalization ability and high reliability on unseen data. By calculating the relative error between the predicted values and the true values, it could be found that the prediction errors of the 77 training samples were all below 10%, and the error percentage between the predicted values and the true values output by the 10 validation samples was all below 10% (Figure 5), which belonged to a relatively low error. This reflects that the prediction level and reliability of the prediction model established based on particle swarm optimization are relatively good, basically in line with the prediction of the release amount of Cu.

**Table 4:** Evaluation metrics of PSO-SVM on training and ﻿test sets.

Model	Evaluation index	Training set	Test set
PSO-SVM	RMSE	0.0232	0.0762
	MAE	0.0220	0.0525
	R^2^	0.9906	0.9045

## 4 Discussion

This study was conducted under environmental simulation in the workplace, as the CuO in antibacterial fibers act by releasing metal ions such as Cu^2+^ (43), and the chemical properties of CuO determine its release mechanism. Previous studies have shown that CuO can generate Cu^2+^ through dissolution or surface ion exchange in weakly acidic and humid environments (44, 45). Additionally, the presence of oxygen vacancies and surface Cu species in CuO-based materials has been shown to influence their reactivity and dissolution behavior (46), further supporting the observed release trends under varying environmental conditions. At the same time, there may be other components (such as additives or heteroatoms) in the fibers that affect the bonding strength between CuO and the fibers. When the fibers swell or undergo chemical interactions, it will accelerate the dissolution of CuO.

Meanwhile, higher temperatures can enhance the surface fluidity of polymer fibers, and even cause polymer chain breakage and surface cracks, thereby promoting the desorption and dissolution of doped CuO particles (47). For CuO materials themselves, ﻿the study has shown that UV irradiation can accelerate the release of Cu^2+^ from CuO particles into ﻿aqueous solution (48), but experimental data shows that radiation has limited effect on the amount of Cu released. ﻿The previous study has indicated that this phenomenon suggests that the effects of radiation aging can damage the fiber structure (49) and lead to the release of Cu in the facepiece. This study speculates that irradiance mainly indirectly affects Cu release by accelerating fiber aging, but this effect is far less than the driving effect of temperature changes on release. When the exposure time of temperature increases, the experimental results show that the release of Cu in disposable facepiece increases significantly, and when the temperature increases, the release of Cu in disposable facepiece increases significantly. One study showed that when the temperature rose from 15 °C to 40 °C, the number of micro plastics released from disposable facepiece increased from 1,043 to 2,940 items/(piece·d), nearly tripling (47). ﻿From the literature, it can be inferred that the release of Cu in ﻿disposable facepiece is mainly affected by temperature. Under the combined action of irradiance and temperature, especially when the temperature exposure time increases, the release of Cu in the disposable facepiece is more than 10 μg, and some even exceed 20 μg. In terms of worker exposure levels, although the amount of Cu inhaled in the short term is within an acceptable safe range, approximately 20 μg of Cu may be inhaled under extremely harsh environmental conditions. ﻿When the release of Cu in CuO disposable facepieces is 20 μg, it accounts for approximately 3% of the total Cu content in disposable facepieces (5). However, it should be noted that previous studies have reported that CuO particles can cause lung inflammation and systemic toxicity through oxidative stress pathways after inhalation (50, 51). Additionally, exposure to elevated metal concentrations, including Cu, has been linked to adverse reproductive health effects, such as sperm DNA damage (52), further emphasizing the need for careful monitoring of occupational Cu exposure.

In this study, ML was utilized to predict the exposure level of ﻿workers. It was found in the training set that SVM has significant advantages in small sample learning due to its good generalization ability (53). However, BPNN is slightly inferior to SVM. This may be because BPNN is prone to falling into local optima when facing small samples and complex nonlinear relationships (54). At the same time, previous studies have shown that SVM performs better than BPNN and RF in predicting the exposure level of substances (55, 56). And data augmentation is utilized to expand the data, and PSO is used to optimize SVM, thereby improving the regression accuracy of the model on the training set. Compared with previous studies, SVM prediction performance is improved after adjusting the parameters of the model (55). Moreover, better generalization performance is also achieved on the test set through the optimized parameters, thereby reducing the risk of SVM overfitting to a certain extent.

Finally, this study has systematically evaluated and predicted the release of Cu in CuO disposable facepiece and its workers’ exposure level as far as possible under the existing technology and time. During the experimental design phase, we conducted a comprehensive literature search and screening, ultimately incorporating all recognized and quantifiable major environmental factors into the model. Although limited by cognition and objective conditions, it is still impossible to exhaust all unknown factors, but the existing evidence is enough to suggest that the Cu released by disposable facepiece has potential risks to workers’ health that cannot be ignored. This study provides a scientific basis for the evaluation and prediction of Cu in CuO disposable facepiece.

## 5 Conclusion

﻿This study has found that there are significant differences in the release of Cu from CuO facepieces across various work scenarios, particularly in harsh working environments where the release of Cu increases significantly, potentially posing risks to the occupational health of workers. Therefore, measuring the release amount of Cu from disposable facepieces containing Cu in different work scenarios and determining the exposure level of workers have been essential to ensure occupational health. To this end, this study has constructed prediction models based on BPNN, RF, and SVM, and has compared the predictive performance of the three models. The results have shown that the SVM model performs well on the training set, but there was a certain degree of overfitting on the test set. To further enhance the generalization ability of the model, this study has used the PSO algorithm to optimize the hyperparameters of the SVM model. The optimized PSO-SVM model has exhibited extremely high fitting accuracy on the training set, with an RMSE of 0.0232, an MAE of 0.0220, and an R² of 0.9906; on the test set, the PSO-SVM model has shown good predictive performance with an RMSE of 0.0762, an MAE of 0.0525, and an R² of 0.9045. In summary, the SVM model based on PSO optimization has shown high accuracy and reliability in predicting the release of Cu from facepieces, providing an effective tool for the occupational health assessment of workers.

## Data Availability

The original contributions presented in the study are included in the article/Supplementary material, further inquiries can be directed to the corresponding author/s.
