# Supplementary material for: ﻿Assessment and prediction of copper release amount from copper oxide facepieces
Source: Front Public Health. 2025 Sep 25;13:1664838. doi: 10.3389/fpubh.2025.1664838 (PMC12507794; doi:10.3389/fpubh.2025.1664838)
Supplement: Supplementary file 1 [file Table_1.docx]

Table S1 shows the release amount of Cu from CuO disposable facepiece under the combined influence of temperature, temperature exposure time, irradiance and irradiance exposure time by the ultrasonic bath treatment.

Table S1. The release amount of copper in the facepiece under various factors

| Sample Number | Temperature/℃ | Temperature time /h | Irradiance /µW/cm^2^ | Irradiation time /h | Absolute content /ug |
| --- | --- | --- | --- | --- | --- |
| 1 | 30 | 2 | 75 | 2 | 9.82 |
| 2 | 30 | 2 | 75 | 5 | 7.25 |
| 3 | 30 | 2 | 75 | 8 | 8.88 |
| 4 | 30 | 2 | 130 | 2 | 9.19 |
| 5 | 30 | 2 | 130 | 5 | 8.75 |
| 6 | 30 | 2 | 130 | 8 | 9.40 |
| 7 | 30 | 2 | 185 | 2 | 9.05 |
| 8 | 30 | 2 | 185 | 5 | 9.80 |
| 9 | 30 | 2 | 185 | 8 | 10.70 |
| 10 | 30 | 5 | 75 | 2 | 13.04 |
| 11 | 30 | 5 | 75 | 5 | 17.48 |
| 12 | 30 | 5 | 75 | 8 | 23.65 |
| 13 | 30 | 5 | 130 | 2 | 15.85 |
| 14 | 30 | 5 | 130 | 5 | 18.37 |
| 15 | 30 | 5 | 130 | 8 | 19.54 |
| 16 | 30 | 5 | 185 | 2 | 17.91 |
| 17 | 30 | 5 | 185 | 5 | 21.78 |
| 18 | 30 | 5 | 185 | 8 | 18.41 |
| 19 | 30 | 8 | 75 | 2 | 17.23 |
| 20 | 30 | 8 | 75 | 5 | 12.57 |
| 21 | 30 | 8 | 75 | 8 | 18.48 |
| 22 | 30 | 8 | 130 | 2 | 12.90 |
| 23 | 30 | 8 | 130 | 5 | 17.30 |
| 24 | 30 | 8 | 130 | 8 | 14.59 |
| 25 | 30 | 8 | 185 | 2 | 18.35 |
| 26 | 30 | 8 | 185 | 5 | 17.38 |
| 27 | 30 | 8 | 185 | 8 | 14.63 |
| 28 | 50 | 2 | 75 | 2 | 11.77 |
| 29 | 50 | 2 | 75 | 5 | 15.88 |
| 30 | 50 | 2 | 75 | 8 | 13.47 |
| 31 | 50 | 2 | 130 | 2 | 19.57 |
| 32 | 50 | 2 | 130 | 5 | 19.33 |
| 33 | 50 | 2 | 130 | 8 | 16.38 |
| 34 | 50 | 2 | 185 | 2 | 12.96 |
| 35 | 50 | 2 | 185 | 5 | 16.34 |
| 36 | 50 | 2 | 185 | 8 | 13.63 |
